# CRADLE-5: a stepped-wedge type 2 hybrid implementation-effectiveness cluster randomised controlled trial to evaluate the real-world scale-up of the CRADLE Vital Signs Alert intervention into routine maternity care in Sierra Leone—study protocol

**DOI:** 10.1186/s13063-023-07587-4

**Published:** 2023-09-15

**Authors:** Alexandra E. Ridout, Francis L. Moses, Simren Herm-Singh, Cristina Fernandez Turienzo, Paul T. Seed, Venetia Goodhart, Nicola Vousden, Betty Sam, Mariama Momoh, Daniel Kamara, Katy Kuhrt, Sorie Samura, Candace Beoku-Betts, Alice Hurrell, Kate Bramham, Sartie Kenneh, Francis Smart, Lucy Chappell, Jane Sandall, Andrew Shennan, on behalf of CRIBS Collaborative Group

**Affiliations:** 1 Department of Women and Children’s Health, School of Life Course and Population Sciences, King’s College London, Westminster Bridge Road, London, SE1 7EH UK; 2 Reproductive Health and Family Planning, Ministry of Health and Sanitation, Freetown, Sierra Leone; 3 Welbodi Partnership, Freetown, Sierra Leone; 4 National Perinatal Epidemiology Unit, University of Oxford, Oxford, UK; 5 National Emergency Medical Service, Ministry of Health and Sanitation, Freetown, Sierra Leone

**Keywords:** CRADLE, Sierra Leone, Low- and middle-income countries, Pre-eclampsia, Scale-up, Shock, Maternal mortality, Blood pressure, Complex intervention, Stepped-wedge cluster trial

## Abstract

**Background:**

The CRADLE Vital Signs Alert intervention (an accurate easy-to-use device that measures blood pressure and pulse with inbuilt traffic-light early warning system, and focused training package) was associated with reduced rates of eclampsia and maternal death when trialled in urban areas in Sierra Leone. Subsequently, implementation was successfully piloted as evidenced by measures of fidelity, feasibility and adoption. The CRADLE-5 trial will examine whether national scale-up, including in the most rural areas, will reduce a composite outcome of maternal and fetal mortality and maternal morbidity and will evaluate how the CRADLE package can be embedded sustainably into routine clinical pathways.

**Methods:**

CRADLE-5 is a stepped-wedge cluster-randomised controlled trial of the CRADLE intervention compared to routine maternity care across eight rural districts in Sierra Leone (Bonthe, Falaba, Karene, Kailahun, Koinadugu, Kono, Moyamba, Tonkolili). Each district will cross from control to intervention at six-weekly intervals over the course of 1 year (May 2022 to June 2023). All women identified as pregnant or within six-weeks postpartum presenting for maternity care in the district are included. Primary outcome data (composite rate of maternal death, stillbirth, eclampsia and emergency hysterectomy) will be collected. A mixed-methods process and scale-up evaluation (informed by Medical Research Council guidance for complex interventions and the World Health Organization ExpandNet tools) will explore implementation outcomes of fidelity, adoption, adaptation and scale-up outcomes of reach, maintenance, sustainability and integration. Mechanisms of change and contextual factors (barriers and facilitators) will be assessed. A concurrent cost-effectiveness analysis will be undertaken.

**Discussion:**

International guidance recommends that all pregnant and postpartum women have regular blood pressure assessment, and healthcare staff are adequately trained to respond to abnormalities. Clinical effectiveness to improve maternal and perinatal health in more rural areas, and ease of integration and sustainability of the CRADLE intervention at scale has yet to be investigated. This trial will explore whether national scale-up of the CRADLE intervention reduces maternal and fetal mortality and severe maternal adverse outcomes and understand the strategies for adoption, integration and sustainability in low-resource settings. If successful, the aim is to develop an adaptable, evidence-based scale-up roadmap to improve maternal and infant outcomes.

**Trial registration:**

ISRCTN 94429427. Registered on 20 April 2022.

## Introduction: background and rationale {6a}

In Sierra Leone (SL), the reduction of maternal and neonatal morbidity and mortality are high priorities of the health sector, in line with the third Sustainable Development Goal [1]. Despite an almost 40% reduction in the maternal mortality ratio (MMR) from 1165 per 100,000 in 2013 to 717 per 100,000 live births in 2019 [2], SL still has one of the highest MMR in the world. One in thirty-one women die during childbirth [3], with a 40 to 60 percent higher risk for teenage mothers [4]. Perinatal mortality is high (34 per 1000 live births), with a stillbirth rate of 24 per 1000 live births [3]. Infants whose mothers die are up to ten times more likely to die within the first two years of life [5].

Data from the CRADLE-3 trial [6] correlate with national statistics; 76% of maternal deaths are caused by haemorrhage, hypertension and sepsis [7]; all of which are marked by abnormalities in blood pressure (BP) and heart rate (HR). If deteriorating vital signs are detected early, maternal morbidity and mortality are preventable with simple, cost-effective interventions that are widely available in SL [6, 8]. However, there are often disparities in access to vital sign assessment and skilled intervention, and delays in delivery and escalation of care [9, 10], varying by geographical area and socio-demographic characteristics.

Vital sign measurement is an international standard part of maternity care. The CRADLE Vital Sign Alert (VSA) device is a hand-held, semi-automated, low-cost BP and HR measuring device with a built-in traffic-light warning system that has been especially developed for use in low-income settings during pregnancy and the postpartum period [11]. The device has additionally been validated as accurate for use in women with preeclampsia [12] and in those who develop low BP from sepsis or haemorrhage [13].

The CRADLE-3 trial [14] investigated the introduction of the CRADLE intervention into routine maternity care in ten clusters across Africa, India, and Haiti. Freetown, SL’s urban capital city, reported a 40% reduction in the primary outcome (at least one of eclampsia, emergency hysterectomy, and maternal death) [odds ratio (OR) 0.60 (95% CI 0.50–0.72), *p* < 0.001] and a 60% reduction in maternal death (risk ratio (RR) 0.37 [95% CI 0.25 to 0.55], *p* < 0.0001) [6]. This trial confirmed that deaths and associated morbidity are avoidable with appropriate triage and escalation. A concurrent process evaluation across all CRADLE-3 trial sites found considerable variation in the implementation, reach, adoption and context; analysis in individual countries was recommended [15].

**Fig. 1:**
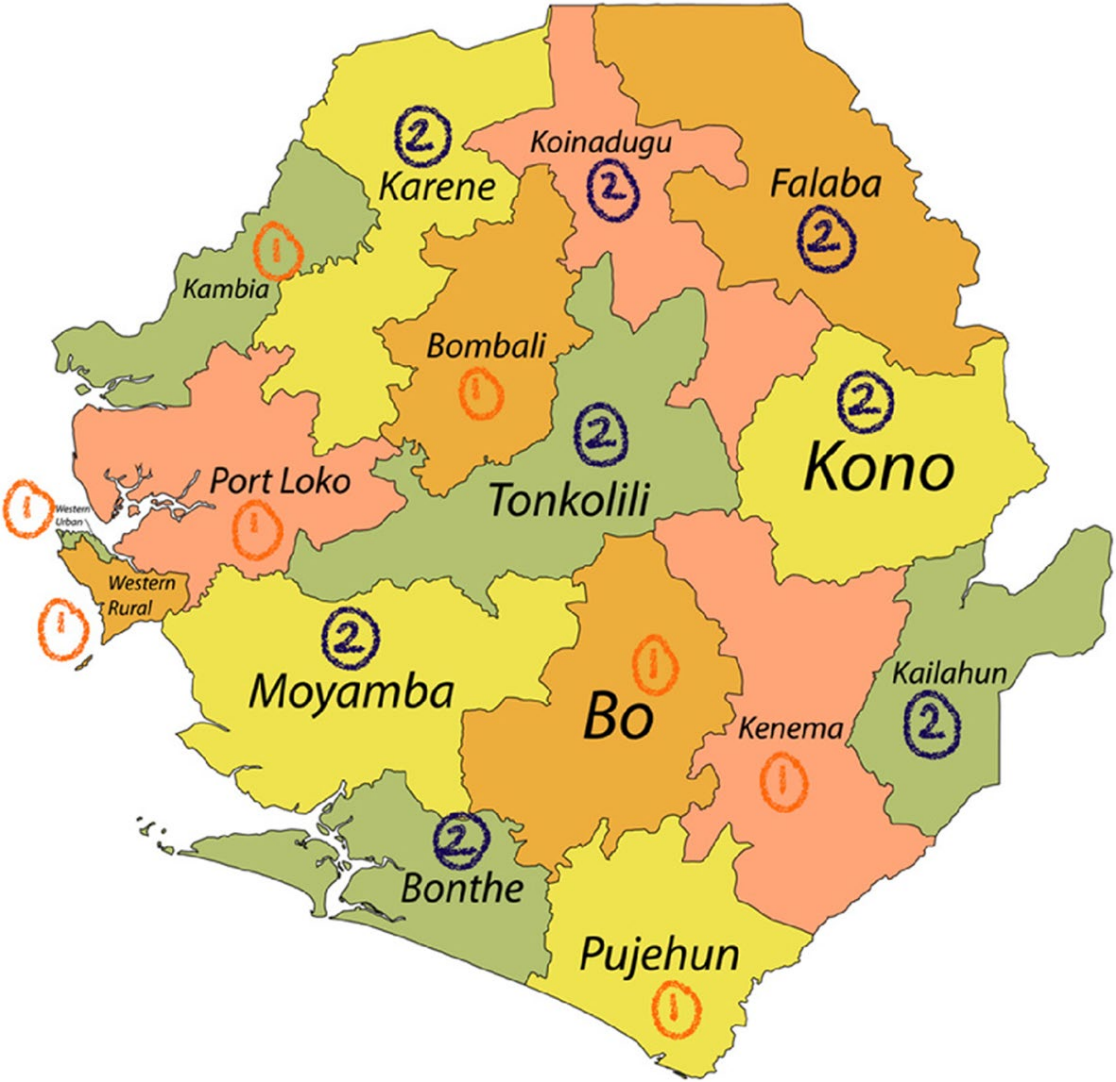
Map of Sierra Leone. Districts which already have the CRADLE intervention are marked (1) and districts included in the CRADLE-5 trial are marked (2). Attribution: Karte: NordNordWest, Lizenz: Creative Commons by-sa-3.0 de

This evidence base combined with an increased focus on timely maternity triage and escalation led the SL Ministry of Health and Sanitation (MoHS) to advocate for the pilot of the CRADLE intervention across eight out of the county’s sixteen districts between March 2020 and January 2021 (funded by the UK Foreign, Commonwealth & Development Office, the World Health Organization and UNICEF). Full results will be published separately, but in summary, 160 CRADLE Champions and 40 Master Trainers were trained, and 2800 CRADLE devices and 1200 large cuffs distributed. The rollout reached 716 peripheral health unit (PHU) facilities providing maternity care, and rolled out to 2031 frontline healthcare staff, showing high levels of feasibility and acceptability. Strong acceptance from frontline healthcare staff and robust evidence prompted buy-in and advocacy by the MoHS in Sierra Leone for horizontal scale-up of the CRADLE intervention to the rest of the country (Fig. 1).

Our set goal is the scale up of the CRADLE intervention to improve access to quality and timely maternity assessment to enable appropriate care for pregnancy hypertension and shock related to obstetric haemorrhage and infection. The remaining eight districts are rural and provide the opportunity to evaluate the impact of CRADLE in this setting, where appropriate triage and referral may have greater impact on outcomes.

This protocol describes the stepped-wedge cluster-randomised controlled trial to evaluate both the clinical impact and scale-up of the CRADLE VSA and training package in Sierra Leone. In-depth context and system specific pilot work highlighted district-specific adaptations and implementation strategies which, alongside our theory of change workshops, has substantially informed the CRADLE-5 trial scale-up strategy [9]. Our aim is to ultimately develop an adaptable, evidence-based roadmap for scale-up in order to achieve sustainable impact on maternal and fetal outcomes. We have been guided by four key principles: systems thinking; a focus on sustainability; the need to determine scalability; and respect for gender, equity and human rights principles [16].

## Hypotheses

We hypothesise that integrating the use of the CRADLE VSA device into routine maternity care and scaling-up focused CRADLE training to the remaining half of SL will increase *availability* of vital signs monitoring equipment, *engage* and *empower* healthcare workers, communities and women to understand the importance of vital sign measure, and enable *reassurance* or alert users and women to when results are abnormal, ensuring prompt life-saving *communication*, *action* and/or *referral* for both mother and unborn baby from the three leading causes of maternal death: obstetric haemorrhage, sepsis and pregnancy hypertension.

## Objectives {7}

The primary objective of the trial is to determine whether real-world scale-up of the CRADLE VSA device and focused education package to community and facility level maternity care in SL is sustainably adopted and reduces a composite of (all-cause) maternal mortality or major morbidity and fetal loss by ≥ 20%.

The trial is complemented by a simultaneous process and scale-up evaluation informed by Medical Research Council guidance [17] and WHO ExpandNet tools [16]. The specific goals of the process and scale up evaluation are to (1) analyse how, and to what extent, the CRADLE intervention has been implemented, (2) assess how the different contexts can influence the implementation process and scale-up strategies and (3) assess the process and sustainability of the scale-up. This will include an evaluation of cost-effectiveness.

## Trial design {8}

### Feasibility phase: January 2022–April 2022

The trial was preceded by a feasibility phase. The eight main trial districts collected primary outcome data to evaluate the methods of data collection and to inform the randomisation programme and sample size calculation. The results were used to optimise the final CRADLE-5 protocol including training materials and implementation strategies for the main trial.

### Definitive stepped-wedge cluster-randomised controlled trial: May 2022 to June 2023

The CRADLE-5 trial is a pragmatic, mixed-methods, multi-district, stepped-wedge cluster-randomised controlled trial of the scale-up of the CRADLE intervention (CRADLE VSA device and CRADLE training package) into routine maternity care settings in SL and will be reported in line with the CONSORT stepped wedge cluster randomised trials update [18]. It will be accompanied by a concurrent process and scale-up evaluation between January 2022 to June 2023.

## Methods: participants, interventions, and outcomes

### Study setting {9}

The study is taking place in SL, a country on the southwestern coast of Africa with a population of 8.8 million [19].

SL is a least developed country according to the OECD list [20] and has one of the highest mortality rates in the world, with a high burden of communicable and non-communicable diseases. It struggles to provide basic universal health coverage, portable water and sanitation. All public health facilities offer free healthcare services to pregnant women, lactating mothers and children below the age of five; however, 72% of women report at least one serious problem in accessing health care for themselves when they are sick; the most commonly cited problem is getting money for treatment (67%) [2]. SL suffers an inadequacy of human resource and diagnostic readiness for health. Nineteen per cent of health facilities have no BP device, and 82% of maternal deaths occurred in a health facility [10]. Latest figures report 0.7 doctors and 7.5 nurses and midwives per 10,000 population [21], well below WHO’s health workforce targets for UHC and SDGs of 23 doctors, nurses and midwives per 10,000. Suboptimal maternal healthcare coverage contributes to preventable deaths, disability and high out-of-pocket spending in a country with an incipient financial crisis, exacerbated by the 2014 Ebola epidemic and further weakened by the COVID-19 pandemic. The capacity and resources to develop and implement national strategies and to produce robust needs-based staffing remain a priority. Our proposed interventions will be evaluated for impact on maternal and fetal outcomes, and cost-effectiveness to take all aspects into account.

The country is divided into five administrative regions (Northern, Southern, Eastern, North-western, and Western) which are further subdivided into 16 districts. Each trial cluster is made up of a specific district in SL; the following eight districts, which have not yet received the CRADLE intervention, were included in the trial: Bonthe, Falaba, Karene, Kailahun, Koinadugu, Kono, Moyamba and Tonkolili. From our selected districts, populations range between 200,781 (Bonthe) to 531,435 (Tonkolili). Bonthe is the smallest, covering an area of 3469 km^2^, whereas Koinadugu is 12,121 km^2^.

Each district comprises a secondary hospital health centre with multiple satellite primary peripheral health units in the community. The total number of peripheral health units per district ranges from 50 in Falaba to 109 in Moyamba. The trial will work across all levels of healthcare facility in the country, reaching the most deprived, remote, and rural communities. Seven of the districts comprise of at least one secondary health facility with multiple satellite primary care centres (called peripheral health units in Sierra Leone) that refer into the secondary hospital. One district (Falaba) does not have a secondary hospital, and referrals from its satellite primary care centres go primarily to the secondary hospital in Koinadugu, as well as to other neighbouring districts.

### Eligibility criteria {10}

Eligibility criteria for the clusters included not having been previously exposed to the intervention. All women identified as pregnant or within the 6-week postpartum period, presenting for antenatal, intrapartum or postpartum care in the district within the trial time frame will be included. There are no exclusion criteria.

### Who will take informed consent? {26a}

In accordance with recognised procedures for stepped-wedge cluster trials [18], institutional-level consent and relevant local approvals from district health authorities and hospitals on behalf of the cluster will be obtained at the start of the trial (time point zero), prior to implementation of the intervention at subsequent time points.

### Additional consent provisions for collection and use of participant data and biological specimens {26b}

Written informed consent will be sought from women, healthcare providers and community members participating in qualitative interviews and focus group discussions undertaken as part of the process or scale-up evaluation. A gatekeeper (for example a community chief) will facilitate the team to approach potential participants and a research assistant will discuss the study, give an information sheet and respond to any queries or concerns of those interested in taking part. Up to 24 h will be given to consider participation. The participant information materials and informed consent form to participate in qualitative interviews and focus group discussions are available from the corresponding author on request.

## Interventions

### Explanation for the choice of comparators {6b}

**Fig. 2:**
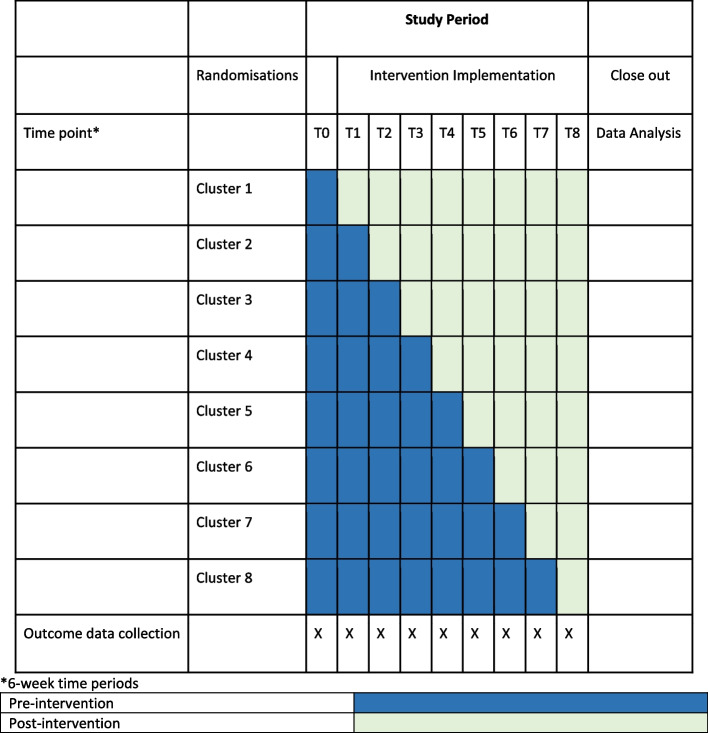
Standard Protocol Items: Recommendations for Intervention Trials (SPIRIT) figure

Prior to transitioning to the CRADLE intervention, all women will be managed according to local guidelines (see https://mohs2017.files.wordpress.com/2018/04/sl-emonc-protocols-and-guidelines-a4-final.pdf to access national guidelines for emergency obstetric and newborn care). None of the districts will receive the intervention at time point 0. One district will be randomly allocated to receive the intervention at time point 1, whilst all the remaining areas contribute to the control at this time point. A second district is randomly allocated to the intervention at time point 2 and so on. Each randomisation cluster will cross over from the control to the CRADLE intervention at six-weekly intervals (see Fig. 2).

At the time each district is randomised to receive the CRADLE intervention, all levels of healthcare providers within that district, involved in the care of pregnant and postpartum women, will get access to the CRADLE VSA device and will be trained using the train-the-trainer model.

### Intervention description {11a}

The CRADLE intervention consists of two components and is described according to the TIDieR checklist [22].

#### Component 1—Microlife® CRADLE Vital Sign Alert device

The Microlife® CRADLE VSA is a hand-held, upper-arm, semi-automated device that measures BP and pulse. The device has undergone extensive testing for accuracy and is one of few BP devices to have been validated as accurate in pregnancy (including pre-eclampsia and hypotension) and in non-pregnant adults [11, 23, 24]. It is cost-effective (less than $20) and can be used effectively by unskilled personnel after minimal training. The device incorporates a traffic-light early warning system that alerts all levels of healthcare provider to abnormalities in vital signs, secondary to obstetric haemorrhage, sepsis and hypertension [23, 25, 26]. The thresholds that trigger the traffic lights were determined through pregnancy-specific prediction studies. The device fulfils the World Health Organization requirements for low-resource settings [24]. Other unique developments suited to low-income countries include a micro-USB charging ability and a “traffic light” early warning system for hypertension and shock (secondary to either obstetric haemorrhage or sepsis). The CRADLE intervention will be incorporated into routine maternity care. All primary and secondary level health facilities in eight districts will be provided with CRADLE VSA devices and emergency obstetric triage training via the Train-the-Trainer model. Devices are allocated per health facility according to health facility size and capacity, with 30% total stock kept at the district stores as buffer stock.

#### Component 2—CRADLE Training programme

A face-to-face training programme will be rolled out to a selected sample of locally selected CRADLE champions, district stakeholders and key maternal health partners in the district headquarter towns by the central CRADLE team, comprising two senior midwives and the program manager. The training package is as described below but can be adapted to meet specific contextual factors. It includes a short, animated video describing the device and how it is used (https://cribs-i.org/professionals/cradle-vsa/microlife-cradle-vsa-user-guide/), an interactive session with the trainees, a reference booklet, a wall poster showing how the device is used and an aide-memoire card attached to the CRADLE device for quick reference. The animated film is in English and has also been translated into Krio, the most commonly spoken language across Sierra Leone. The content of this training material covers: (1) use of the Microlife CRADLE VSA device, (2) basic clinical assessment of pregnant women, (3) understanding pre-eclampsia, eclampsia, obstetric haemorrhage and sepsis and review of local management guidelines and protocols, (4) triggers for treatment and/or referral and familiarisation with local referral pathway, (5) CRADLE VSA device troubleshooting and (6) CRADLE VSA maintenance manual.

Components of the training have been adapted to meet the SL specific contextual needs: the training was expanded to include a more extensive focus on Emergency Obstetric and Newborn Care (EmONC) training as well as an increased focus on community engagement, training facilitation tips, respectful maternity care and a CRADLE maintenance practical session. A training aide in the form of the CRADLE song, to a local popular tune, was integrated.

Champions were encouraged to follow the ten-step training content approach:

1. Explain CRADLE (CRADLE video)
2. Explain the traffic lights
3. Explain the arrows
4. Explain the different scenarios and their management
5. Practical demonstration of how to use the device
6. Explain respectful maternity care and role play to communicate CRADLE results with women
7. Demonstrate how to document the CRADLE results
8. Practical session using the CRADLE observation checklist
9. Explain how to maintain CRADLE and explain the “Replace & Repair” system
10. Sing the CRADLE Song

Following training, the CRADLE champions return to their allocated local community health centres (three or four maximum) and deliver the devices and associated training. Additional CRADLE Champions are selected from the district hospitals to target the higher numbers of staff working within hospital settings. CRADLE Champions are encouraged to conduct the device distribution with involvement and in the presence of key community stakeholders, to raise awareness of the CRADLE intervention and its impact on supporting maternal health and antenatal care delivery at community level.

### Criteria for discontinuing or modifying allocated interventions {11b}

There are no criteria for discontinuing the intervention given it is a validated, accurate device and vital signs measurement is part of standard care during pregnancy and the post-partum period. The CRADLE devices and training materials will continue to be available across the healthcare system at the end of the trial.

### Strategies to improve adherence to interventions {11c}

Several strategies have been employed to promote adherence to the intervention protocol. A theory of change workshop bringing together key stakeholders across community, district management and national levels facilitated discussion around the pathways of change for the CRADLE intervention to improve uptake and quality of maternity services, particularly learning from the pilot phase across the first eight districts. Engagement visits to all eight districts were undertaken prior to the feasibility phase and trial. Two offline mobile phone applications were developed to aid primary outcome and process evaluation data collection; both have integrated Global Positioning System (GPS) geotagging capabilities allowing real time monitoring of data collection and CRADLE training adherence. Three-monthly monitoring trips are undertaken to supervise, monitor and audit data collection of primary and secondary outcomes, in addition to real time online data monitoring. Based on the pilot within the first eight districts, the CRADLE training was refined to include a more in-depth focus on emergency obstetric and newborn care (EmONC), increased community engagement to facilitate patient referral acceptance by stakeholders and increased hospital engagement to support adoption of the CRADLE intervention across multi-disciplinary teams and cadres. Full pilot phase implementation strategies will be published elsewhere.

### Relevant concomitant care permitted or prohibited during the trial {11d}

No routine concomitant care will be withheld during the trial.

### Provisions for post-trial care {30}

As sponsor, King’s College London has specialist insurance policy in place which would operate in the event of any participant suffering harm because of their involvement in the research. However, the CRADLE intervention (i.e. blood pressure and pulse measurement, and training) is an international standard, is of minimal risk and does not adversely affect the rights and welfare of the individual women.

### Outcomes {12}

Analysis of each outcome will be as a proportion of all deliveries during the six-week period within that district, as assessed from routinely collected data.

#### Primary outcome measure

The primary outcome is the rate of a composite of maternal mortality, major maternal morbidity and fetal losses (with no double counting) occurring during pregnancy, labour or within 42 days of delivery, including at least one of:

- Maternal death (all-cause mortality)
- Eclampsia (occurrence of generalised convulsions with increased blood pressure in the absence of epilepsy or another condition predisposing to convulsions)
- Emergency hysterectomy (surgical removal of all or part of the uterus)
- Stillbirth (born without signs of life at or after 28 weeks of gestation)

The primary outcome measures will be assessed by the numbers of events reported at the district (or referral) hospital and the peripheral health units offering the highest level of care (defined as the 20% with the highest level facilities in each district).

#### Secondary outcome measures

Maternal deaths from all-cause mortality will be collected. Additional information will be collected regarding the cause of death, including deaths due to:

1. Any obstetric cause
2. Obstetric haemorrhage
3. Pregnancy-related sepsis
4. Hypertensive disorders of pregnancy

Additional secondary maternal outcome measures will include:

5. Eclamptic fit before hospital admission
6. Eclamptic fit after hospital admission
7. Emergency hysterectomy due to obstetric haemorrhage
8. Emergency hysterectomy due to ruptured uterus
9. Emergency hysterectomy due to other cause

#### Perinatal secondary outcomes

Earlier recognition of pregnancy hypertension during antenatal care should facilitate appropriate timing of delivery and reduction in preventable stillbirth and neonatal morbidity and mortality as a complication of preeclampsia and eclampsia. Conversely, extreme preterm delivery to reduce maternal morbidity and mortality from preeclampsia/eclampsia may result in an increase in neonatal mortality. Therefore, perinatal secondary outcomes will include:

10. Antenatal stillbirth
11. Intrapartum stillbirth
12. Stillbirth beyond 28 weeks’ gestation
13. Early neonatal death (before seven days)
14. Late neonatal deaths (seven to 28 days)

The CRADLE intervention should enable healthcare professionals to recognise abnormal vital signs earlier and manage complications in a timelier manner, with the management of severely compromised pregnant women potentially requiring transfer to higher-level centres for specialist treatment. Normal vital signs should reassure clinicians and reduce inappropriate referrals. Secondary referral outcomes will include an analysis of the National Emergency Medical Service (NEMS) routine database. However, this data will only be analysed if the NEMS data is consistently collected during the entire trial period. Additional outcomes may include:

15. Total number of obstetric referrals
16. Number of referrals related to hypertensive disorders of pregnancy
17. Number of referrals related to eclampsia
18. Number of referrals related to obstetric haemorrhage
19. Number of referrals related to pregnancy-related sepsis

### Process and scale up evaluation of the CRADLE intervention

The process and scale-up evaluation of the protocol intervention will be evaluated via a mixed-methods approach (i.e. routine data, qualitative interviews, focus group discussions, direct observations, reporting forms at baseline and set time points) drawing on the Medical Research Council for complex interventions [17]. We will consider the SL context, our theory of change, the engagement and involvement of stakeholders and understanding uncertainties, adoption and costs. To analyse complex issues in the development of the scale-up strategy during this trial, we will use the ExpandNet/WHO scale-up framework to understand how the CRADLE innovation can being taken to scale in the context of four elements; the resource team, user organisations, scale up strategies and strategic choice areas for managing scale-up [16].

The study population for the evaluation are stakeholders involved in the CRADLE intervention rollout development and implementation process and those involved in the policy dialogues. Stakeholders likely to influence implementation include political leaders, external health actors including NGOs and UN agencies, interest groups including medical and midwifery schools, financial decision makers, beneficiaries and community and family players. The study population for the scale-up evaluation is the target population, i.e. healthcare workers, pregnant / postpartum women living in the relevant districts and communities.

Measures [27] include but are not limited to:

- Fidelity (the number and proportion of facilities that received CRADLE devices, and CRADLE training; the number of devices distributed; the number and proportion of frontline healthcare staff trained in CRADLE; fidelity of CRADLE training);
- Adoption (training to frontline healthcare workers, number and proportion of blood pressures as per CRADLE training);
- Adaptations to the CRADLE intervention and implementation process throughout the trial using self-reported district implementation diaries, evaluating policy dialogue through, e.g. a policy lab, contextual factors and implementation strategies, as well as be explored qualitatively;

Scale-up outcomes of reach, maintenance, sustainability and integration will be assessed through triangulation of findings from different measurement tools.

- Reach will be assessed through GPS-tracked CRADLE training and data collection, pre/post-assessment of blood pressure measurement accuracy (using terminal digit preference); number and appropriateness of referrals before and after CRADLE intervention; timely and accurate escalation of care for women with eclampsia and shock.
- Maintenance assessment of CRADLE BP measurement will entail a mixed methods pre/post-design audit of CRADLE “repair and replace” strategies; spot checks of training quality over time and care pathways will be interrogated through interviews with implementers and the local and national teams across timepoints to assess how CRADLE implementation has improved or deteriorated across its components.
- Sustainability will be assessed through reviewing minutes of quarterly stakeholder meetings focused on device maintenance, ongoing training and adoption across different stages of the trial and policy mapping. The policy map will be filled in to reflect change at local and national level. Both will generate a policy timeline on maternity care, to assess how the context influences CRADLEs activities, and vice versa.
- Progress on integration will be explored through experiences of care pathways for women themselves, healthcare staff and local community stakeholders and ways in which roadmap actions (including interventions, programmes and reforms) should and have been expanded (e.g. integration into national and partner training, procurement and supportive supervisions).

We will explore relationships between process outcomes and the primary outcome to examine whether the effects of the CRADLE intervention differ by implementation. Qualitative work will add deeper understanding of changes in implementation, experiences of the CRADLE package and potential mechanisms to explain initial quantitative results. A combination of both quantitative and qualitative data will be needed to answer our research questions.

### Participant timeline {13}

The participant timeline is presented in Fig. 2.

### Sample size estimation {14}

Sample size estimation has been provided by the CRADLE senior statistician, using Stata version 13.1 and the methods of Hemming and Girling [28]. The power of the study will depend on three factors: (1) the design (eight groups, six-weekly time periods, around 1000 deliveries/period/centre), (2) the event rate and treatment effect (5% falling to 4%) and (3) the data structure, specifically the intraclass correlation coefficient (ICC) and cluster autocorrelation (CAC) coefficients.

**Fig. 3:**
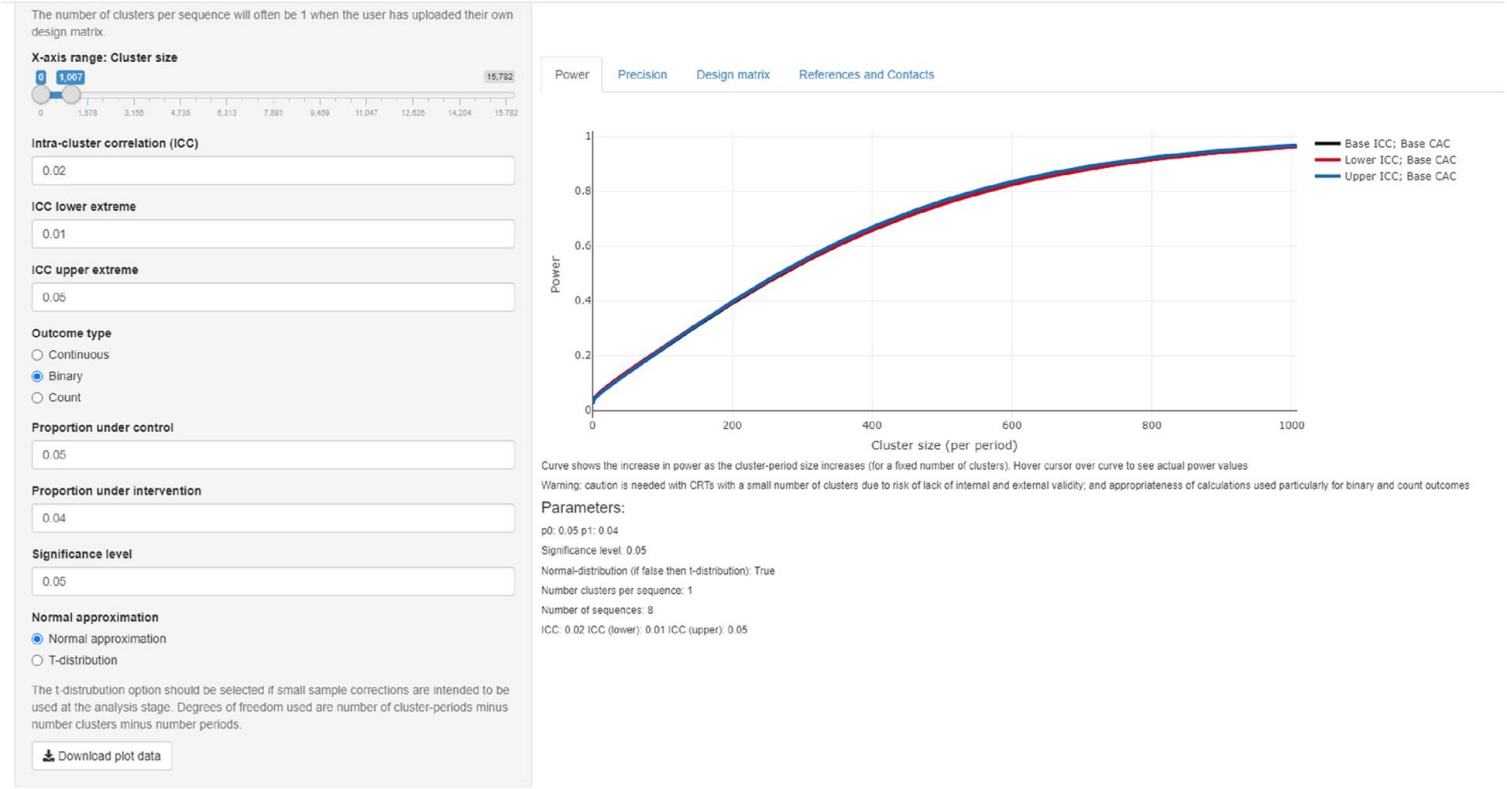
Demonstration modelling of cluster size calculation if ICC 0.01, 0.02 and 0.5 with CAC = 1

Sample size calculations for longitudinal cluster randomised trials, such as our proposed stepped-wedge trials, necessitate the estimation of the correlation structure underlying the study. This entails assessing both the within-period intra-cluster correlations, which differ from conventional intra-cluster correlations due to their reliance on the period, and the cluster autocorrelation coefficients to account for correlation decay. For the purposes of the power calculation, we assume that there are up to 1000 deliveries per district per six weeks (half the 2000 we consider likely), across eight districts, each observed for 12 months. It is difficult to estimate more accurately at present, as we have no suitable multi-centre, multi-time period data in a single low-income country. However, if the ICC is suitably low and the CAC is suitable high, at the time of analysis, we do not predict any difficulties. We will obtain this information from our observational pilot data gathered in January to May 2022 in all eight districts of the study. Feasibility data suggests the CAC is likely to be 1. Therefore, even with an extremely high intra-cluster correlation of 0.5, power would be close to 100%. We have used https://clusterrcts.shinyapps.io/rshinyapp/ for the calculations. As per Fig. 3, with a CAC of 1, power will be more than adequate with 1000 women per month per centre.

We intend to consider whether the treatment effect can be linked to the extent and quality of the implementation of the intervention in each district, as measured by our implementation and scale-up metrics. An absolute difference of 0.25% in the treatment effect between districts would clearly be important. Given the high power for the main treatment effect, we anticipate that we will have more than adequate power to detect such a difference.

### Recruitment {15}

The intervention is delivered at the level of the district, rather than the level of the individual woman, and standard care will encompass exposure to the intervention [18].

## Assignment of interventions: allocation

### Sequence generation {16a}

The unit of randomisation in our trial is the cluster (a district), rather than individual women. Two clusters, Falaba and Bonthe, were paired before randomisation. The districts will first be allocated random area numbers, and the sequence of timing for receiving the CRADLE intervention will then be determined by computer-generated random numbers by the CRADLE statistician.

### Concealment mechanism {16b}

The districts will be masked until they are informed of their allocation four to six weeks prior to their implementation date to give sufficient time for training and distribution of devices to be arranged.

### Implementation {16c}

A new district will come on board every 6 weeks, at which point all facilities will have received their devices, and all frontline healthcare workers will have been trained in the CRADLE intervention model, as described above. This is a pragmatic trial and any deviation from the implementation plan will be recorded.

## Assignment of interventions: blinding

### Who will be blinded {17a}

The nature of the CRADLE intervention is such that blinding of women and healthcare providers cannot be achieved. However, study assignment will be masked to the CRADLE statistician and the researchers who will analyse the data.

### Procedure for unblinding if needed {17b}

The trial is not blinded; therefore, there is no unblinding procedure.

## Data collection and management

### Plans for assessment and collection of outcomes {18a}

Primary outcome data will be collected using a bespoke phone application-based electronic case report form (eCRF). District research officers were provided tablets and data allowance for this purpose. When possible, real-time online data will be uploaded directly into the eCRF, with outcomes therefore recorded directly onto the trial database. The application has been designed to have an offline function given the low availability of internet in many of SL’s rural areas. The outcomes will then upload directly into the study’s electronic database when internet becomes available.

All primary outcomes are robust and meaningful clinical endpoints that are unambiguous and therefore feasible to collect. We have chosen a composite as powering a trial for maternal death alone would require a prohibitively large sample size. We aim to demonstrate the true impact of the intervention across the health system by collecting mortality and morbidity data from hospitals and peripheral health units offering the highest level of care (20% sample. Women presenting to the peripheral health unit and referred to the hospital will be noted, to avoid double counting. Outcomes occurring elsewhere are not included.

The date at first presentation to health facility will be used to determine the period in which a woman experiencing a primary event is analysed in. For example, if a woman presents to the health facility in period one but goes on to be referred to the hospital and suffers an eclamptic fit during period two, she will be analysed within the period one cohort. This is because the district may have received the CRADLE intervention at the beginning of period two; the woman would not have been exposed to the intervention at the time of her initial presentation.

### Plans to promote participant retention and complete follow-up {18b}

Outcome data will be collected by the district research officer (DRO), with the principal investigator (PI) or a nominated deputy providing a second sign-off for all primary outcomes. Consistency and quality of source data will be monitored by the DRO at each area (ongoing). The MedSciNet database allows for extensive monitoring and query processing features, as well as a comprehensive alerting system to identify missing data. Fields including ‘limits’ will be used to avoid entry of erroneous data. The CRADLE-5 trial management team will perform a number of validation checks to verify validity and completeness. Twenty-five per cent of hospital source data will be validated by the central CRADLE team, and this will be reviewed bi-monthly during the visits by the programme manager and/or research assistant to each of the trial areas, to check for transcription errors. It will be possible to review the audit trail at individual data-point levels. Training in the trial protocol will be delivered to teams centrally (before recruitment begins) and on site in the field, to ensure staff are confident and competent to collect outcome data and quality, accuracy and completeness of data collection is ensured.

### Data management {19}

All data uploaded on the MedSciNet database will be automatically stored and backed up. Collection and storage of clinical data in the database will be governed by the UK Data Protection Act 1998. All MedSciNet data is stored on high-capacity servers that are operated by an external company. Servers are stored in locked rooms, with system monitoring 24/7, physical surveillance and surveillance cameras. A tape backup system is used for backing up the database. The MedSciNet database will remain accessible for 1 year following completion of the main trial. A copy of this will then be kept on the KCL server for 20 years following the trial completion date, in accordance with the KCL Data retention schedule. Consent forms for any qualitative aspects of the research will be kept in files in secure areas at the central site (Welbodi Partnership office, Freetown, Sierra Leone). Any personal contact information will be held on a local database kept in a locked environment, after gaining written informed consent from trial participants. Only the research assistant, the local trial project manager and the UK-based trial coordinator will have access to these. All paper documents will be stored securely and kept in confidence in compliance with the UK Data Protection Act 1998.

### Confidentiality {27}

Anonymised data will be collected by the district research officer, under the supervision of the trial coordinator. All participants will be given a unique trial identifier and no personal information will be entered into the clinical trial database or sample database. Where possible, all anonymised data will be collected directly onto the secure, online database (MedSciNet). If the low-resource nature of the environments where we will be collecting the data (e.g. very rural health clinics) means this is not possible, the data will be stored anonymously within a password protected secure phone application. This removes any need for paper-based source data collection tools and ensures confidentiality is maintained.

### Plans for collection, laboratory evaluation, and storage of biological specimens for genetic or molecular analysis in this trial/future use {33}

No biological specimens for genetic or molecular analysis will be collected during this trial.

## Statistical methods

### Statistical methods for primary and secondary outcomes {20a}

This is a type 2 hybrid implementation-effectiveness cluster randomised controlled trial in a stepped-wedge design that will allow us to simultaneously test our clinical intervention in the form of the CRADLE device and the effectiveness of our implementation strategy. This design is based on those described by Hussey and Hughes [29] and Curran et al. [30].

For our primary analysis method, we will use a new approach described by Thompson et al. [31, 32]. For this non-parametric within-period method, Monte Carlo permutation tests for stepped wedge trial designs are employed to calculate the confidence intervals and *P*-values. This is implemented in the Stata command “swpermute” [31]. Based on current statistical theory, this will provide the most accurate and reliable estimates of the intervention effect.

Thompson et al. [31] provide a non-parametric within-period method for correcting the *P*-values (and therefore the standard errors) for a valid regression model. The regression model (binomial, with a log link) that we propose to use will estimate the risk ratio for the effect of the planned intervention. It will include fixed effects for separate linear trends in each region, with a single step at the time the intervention is introduced into the district. As the method is robust to how the correlation structure is specified, further allowances for the data structure, as used in multi-level modelling, are not needed. Odds ratios will be given if models fail to converge when estimating risk ratios. Effects of treatment on primary outcomes will be estimated with 95% confidence intervals and significance at *P* < 0.05. As the primary endpoint is a composite, all the components will also be analysed in the same way. The effect of the intervention on primary and secondary outcomes will be analysed using multilevel logistic regression with random effects of centre and adjustments for fixed categorical effects of time. In addition, we will adjust for the availability of six common drugs: magnesium sulphate (MgSO_4_), ampicillin, gentamycin, metronidazole, oxytocin, and misoprostol, varying with time and district.

We have chosen a composite as powering a trial for maternal death alone would require a prohibitively large sample size. These outcomes are robust and meaningful clinical endpoints that are unambiguous and therefore feasible to collect. Improvements in these outcomes would also represent a success for the CRADLE intervention and will be presented across all districts pre- and post-intervention with adjusted risk ratios (RRs). If there are other large groups of clinically important causes of death, e.g. early pregnancy complications, these will also be defined.

### Interim analyses {21b}

All analyses will adhere to the intention-to-treat principle, meaning that subjects will be analysed based on the treatment option they were assigned to at the centre when the event occurred. No additional analyses based on the per-protocol principle will be conducted.

### Methods for additional analyses (e.g. subgroup analyses) {20b}

We will not be able to collect detailed information (for example breakdown by age or parity) on the total number of deliveries in each district so no subgroup analysis will be possible. No additional analyses based on the per-protocol principle are planned.

### Methods in analysis to handle protocol non-adherence and any statistical methods to handle missing data {20c}

We will make every attempt to get complete data in every district throughout the trial period. In the event of extreme circumstances, such as civil unrest, catastrophic weather events or major epidemic, it is possible that data collection from a specific district may not be feasible for one or more time periods. If such circumstances arise, all valid data will be analysed using the previously mentioned model.

### Plans to give access to the full protocol, participant-level data, and statistical code {31c}

The datasets used and/or analysed during the current study can be made available by the corresponding author upon reasonable request and in agreement with the research collaboration and data transfer guidelines of KCL.

## Oversight and monitoring

### Composition of the coordinating centre and trial steering committee {5d}

The trial will be supported by the Project Management Group (PMG), who includes clinical research fellows, research assistants, DROs and the CRADLE programme manager who will run programme implementation and provide organisational support, particularly to the district research officers and district health teams. The PMG will convene every month, with all co-investigators convening every 3 months to review progress, troubleshoot and plan strategically. This group will report to the trial steering committee (TSC), which is part of a bigger International Advisory Group that oversees all activities of the different workstreams of the NIHR Global Health Research Group CRIBs. The TSC will include dependent and independent members. Members will be provided with a TSC Charter and will be invited to attend, at least, annual TSC meetings.

### Composition of the data monitoring committee, its role, and reporting structure {21a}

Given the low risks of the CRADLE-5 intervention and the unlikelihood of critical safety concerns directly related to implementing the intervention, the trial will not have a data monitoring committees nor interim analysis stopping rule.

### Adverse event reporting and harms {22}

Blood pressure and pulse measurement is standard practice as a screening tool in antenatal care and therefore no adverse events or harms are expected.

### Frequency and plans for auditing trial conduct {23}

There are no additional plans for auditing trial conduct apart from those described in other sections.

### Plans for communicating important protocol amendments to relevant parties (e.g., trial participants, ethical committees) {25}

A ‘substantial amendment’ is defined as an amendment to the protocol or any other supporting document that is likely to affect to a significant degree (1) the safety or physical or mental integrity of the subjects of the trial, (2) the scientific value of the trial, (3) the conduct or management of the trial or (4) the quality or safety of any intervention used in the trial. All substantial amendments will be notified to the chief investigator, who will then notify the competent authority. Non-substantial amendments will be recorded and filed. In case amendments concern or affect participants in any way, they will be informed about the changes. If needed, additional consent will be requested and registered. Also, online trial registries will be updated accordingly.

### Dissemination plans {31a}

The results of this research will be disseminated in international peer-reviewed journals. Both positive and negative results will be reported. All attempts will be made to provide women, healthcare workers and community stakeholders a layman summary of the results within the district that were randomised to the CRADLE intervention, as well as informing government and policy makers.

## Discussion

Three quarters of maternal deaths in SL are caused by haemorrhage, hypertension and sepsis [6], all of which cause abnormalities in vital signs. The majority of deaths are preventable if deteriorating vital signs are detected early, enabling simple interventions to be delivered, which are available in SL. However, access to vital signs monitoring equipment and skilled intervention is often limited, especially in rural settings, leading to delays in escalation and delivery of care. Clinical effectiveness of introduction of the CRADLE intervention to improve maternal and perinatal health in rural areas, and integration and sustainability have yet to be investigated. This trial will evaluate whether national scale-up of the CRADLE device combined with focused training reduces maternal and fetal mortality and severe maternal adverse outcomes and will explore strategies for adoption, integration and sustainability of the intervention in Sierra Leone, which will be relevant to other low-resource settings. Developing a robust clinical trial, to address life-threatening maternal health issues, whilst taking into account the reality of the trial environment is challenging, but there is an urgent need to generate evidence specific to the local context and to evaluate interventions where they are most needed. Examples of potential limitations include capacity and engagement of district medical teams to coordinate and deliver sufficient staff refresher training, given multiple active healthcare initiatives across the country, and technical issues related to the CRADLE device, i.e. charging and broken parts, which could limit sustainable integration and impact. However, this trial design has a strong, context specific evidence base [6, 9], with substantial pilot and feasibility work, and is underpinned by several sustained collaborative partnerships in Sierra Leone, including with the MoHS. The trial protocol was developed with strong and enthusiastic in-country advocacy, with top-down and bottom-up involvement and resultant widespread acceptance of the intervention. Strong stakeholder buy-in, achieved through long-term engagement, theory of change workshops and a policy lab has led to integration of CRADLE into other programmes and initiatives including the National EmONC training package and the ‘train-the-trainer methodology galvanises local teams to take ownership at both district and community level. In summary, this intervention has been tailored for delivery to rural populations, who are amongst the most vulnerable, in terms of access to healthcare, with the worst outcomes. The study aims to generate a pragmatic, evidence-based, adaptable road map to reach, impact and empower women and healthcare workers, even in the most challenging settings.

## Trial status

The current CRADLE-5 protocol is version 1.0 (December 2022). The trial received ethical approval from the Office of the Sierra Leone Ethics and Scientific Review Committee on 24th January 2022 and from the King’s College London, UK Research Ethic Committee on 27 January 2022. The trial opened on the 23 May 2022; all outcomes occurring from 23 May 2022 to 1 January 2024 will be included in the analysis.

## Data Availability

The datasets used and/or analysed during the current study will be made available from the corresponding author upon reasonable request.

## Abbreviations

SL: Sierra Leone
MMR: Maternal mortality ratio
BP: Blood pressure
CRADLE: Community blood pressure monitoring in Rural Africa: Detection of underLying pre-Eclampsia
VSA: Vital Signs Alert
MoHS: Ministry of Health and Sanitation
PHU: Peripheral health unit
WHO: World Health Organization
KCL: King’s College London
